# A century of treeline and forest line data for *Betula pubescens* subsp. *czerepanovii* across high elevations in Norway

**DOI:** 10.3897/BDJ.13.e160358

**Published:** 2025-11-10

**Authors:** Ingrid Vesterdal Tjessem, Peter Horvath, Inger Kristine Følling Volden, Adam Eindride Naas, Michal Torma, Anders Bryn

**Affiliations:** 1 University of Oslo, Oslo, Norway University of Oslo Oslo Norway

**Keywords:** elevational change, monitoring, mountain birch, range shift, remapping, resampling, treeline

## Abstract

**Background:**

The global climate is warming, especially in northern regions due to high-latitude amplification. This high-latitude warming leads to range expansion with advancing tree- and forest-lines (TFLs) in the Northern Hemisphere. However, empirical studies can rarely provide a well-documented elevational expansion rate, especially for timescales longer than 40–50 years. This study provides a unique long-term dataset on TFL dynamics of Betula pubescens subsp. czerepanovii in Norway, based on a combination of resampled historical data (n = 319) and new field registrations (n = 447).

Our dataset includes a total of 766 registrations from five counties in Norway. In total, the dataset contains 439 treelines and 327 forest lines, most likely representing the highest recorded TFLs for the region at the given time. For all data, both resampled and new, locality, coordinates, elevation, aspect and spatial uncertainty and the resampling/sampling methods and definitions are provided. The entire material is stored and available for download through the Global Biodiversity Information Facility (GBIF) portal.

**New information:**

This dataset includes newly-resampled TFLs, based on 57–127-year-old registrations. The entries provide elevational changes, georeferenced localities and potential sites for monitoring climate change effects. The entries enable regional analyses of TFL dynamics on intermediate timescales, including the effect of time lags. The material is available for modelling TFL range shifts along the boreal-alpine ecotone. This dataset most likely provides the highest registered Betula pubescens subsp. czerepanovii locations within their specific regions, thus representing the contemporary ecophysiological range limits for the life-form tree. Additional high-elevation TFL sites and localities have been added to make the material suitable for future remapping and monitoring of climatic TFL dynamics.

## Introduction

Global warming affects the world's vegetation, especially at high latitudes (Pauli et al. 2012, Pepin et al. 2015, Rumpf et al. 2018). High-latitude greening and range expansion of shrubs and trees are evident, particularly in temperature-limited ecosystems (Hudson and Henry 2009, Myers-Smith and Hik 2018, Steinbauer et al. 2018) and, in alpine regions globally, forests are expanding upwards (Harsch et al. 2009, Rannow 2013, Bryn and Potthoff 2018, Cazzolla Gatti et al. 2019) and northwards (Hofgaard et al. 2013).

Along the elevational gradient, treelines and forest lines (TFLs) define the two most striking vegetation boundaries. The forest line (FL) marks the transition zone from boreal forest to low-alpine regions and is characterised by a scattered distribution of trees - a boreal-alpine ecotone. The treeline (TL) indicates the uppermost limit for upright tree growth and the transition to treeless alpine areas. Although changes in treelines may occur independently of shifts in forest lines, both serve as life-form height limits and are influenced by the same underlying drivers and effects (including climate, disturbances and edaphic and topographic conditions). Thus, TFLs are commonly regarded as a unified response and, therefore, used as a combined term in this study.

Both TFLs are primarily limited by low temperatures (Holtmeier 2009, Körner 2012), which restricts the establishment and growth of the life-form tree. A global model predicting treeline positions revealed the growing season mean air temperature limit for tree growth to be 6.4°C +/-0.4°C (Paulsen and Körner 2014). However, the trees of > 2 m height included in the model might have been established 50–60 years ago when conditions were cooler, thus, a treeline position which is in balance with climate would likely be found close to a 6°C isotherm (Körner 2021). Studies of historical FLs, based on fossils, indicate a clear reflection of the varying climatic conditions throughout the Holocene (Paus and Haugland 2017). However, as reported by Körner (2021), the climatic TFLs are not in equilibrium with the contemporary climate unless the climate has remained stable for many years. Forest line dynamics lag considerably behind the contemporary climate and, although largely unknown for the Northern mountain birch forests (Rannow 2013), a general time lag between 50-100 years has been suggested (Körner 2012, Körner 2021).

TFLs are also affected by other climatic variables, as well as biological interactions and physical processes (Holtmeier and Broll 2017, Körner and Hoch 2023). The most important process is previous and ongoing land use (Bryn et al. 2013, Tømmervik et al. 2019), which commonly lowers the TL (Speed et al. 2010).

Research from Norway has pointed out how TFL dynamics currently are built on very few and spatially scattered entries (and studies) (Bryn and Potthoff 2017, Bryn and Potthoff 2018). Additionally, outside Norway, there is a strong need for more empirical data documenting TFL dynamics, especially over timescales that extend beyond the 40 to 50 years typically covered by the majority of the ongoing monitoring projects within the Nordic Region (Cudlín et al. 2017).

For the purpose of documenting historical TFL distributions, accounting for potential time-lags and providing data and sites for future monitoring of TFLs in Norway, we have sampled 439 TLs and 327 FLs of Betula pubescens subsp. czerepanovii (N.I. Orlova) Hämet-Ahti [mountainbirch] along the boreal-alpine ecotone in Norway. Of these 766 in-situ registrations, 319 are resampled locations, whereas 447 are new locations (or from the same location as a historical registration, but then in another aspect, thus regarded as new). We have resampled TFLs, based on entries originally sampled in 1887, 1894, 1913–14, 1917, 1919, 1938, 1954, 1961–62 and 1967, all resampled between 2013 and 2023.

## General description

### Purpose

The purpose of the resampling was to study the elevational dynamics of TFLs, with a particular focus on data older than 40–50 years. By resampling previously analogue and non-georeferenced data, we have improved already existing data on TFLs by now making them digitally available. More explicitly, we wanted to identify the new and updated TFL elevations at old localities, measuring (altitude, tree height etc.) georeferencing the localities and, through GBIF, publishing resampled TFL locations of Betula pubescens subsp. czerepanovii from Norway. Additionally, by adding new TFL locations and sites that have not yet been registered, we have provided a dataset suitable for future monitoring of TFLs in Norway.

### Additional information

In total, the data described contains 319 georeferenced entries of resampled TFLs of Betula pubescens subsp. czerepanovii. Of the resampled TFLs, 247 registrations cover two time periods, a first registry and a second resampled registry from the same site. For the remaining 72 remapped georeferenced entries, the data also include a third period.

Spatial uncertainty is provided for all entries and the resampling method is explained. The entries have recently been published (January 2025) on the GBIF server, where they are stored and available for download.

## Sampling methods

### Study extent

The study extent includes mainland Norway.

### Sampling description

The resampling and sampling of new TFLs were conducted by the authors through in-situ field mapping using the Global Positioning System (GPS) for elevation measurements and confined to the same tree-forming species (Betula pubescens subsp. czerepanovii) and exposition as the original entries.

**Literature search for historical TFLs**: The original TFLs were derived by searching published databases, literature and documents. More specifically, we used the Norwegian literature database “Oria” (https://uio.oria.no), which searches through the University of Oslo Library, as well as all the Norwegian Academic Libraries. Our search included key terms like “treeline”, “forest line”, “timberline”, but also Norwegian terms “tregrense”, “skogrense”, “fjellskog”, “fjellbjørk”, as we expected most of the resulting literature to be written in Norwegian. When we came across literature of potential relevance, we manually skimmed through the literature and targeted information where treelines and/or forest lines were explicitly described with elevation (measured with a compass and aneroid barometer as m a.s.l.). Only information separating TLs from FLs was included in the resampling (see Table 1). None of the original datasets provided data that was properly georeferenced, i.e. with XY coordinates (latitude/longitude) and the original data only provided names of the specific mountain alongside its aspect. All the original TFLs were recorded in situ.

**Inclusion criteria for original TLF registrations**:

Data on TFLs from the original authors were only included if the following information was provided:

- precise locality names (name of municipality and mountain/site)
- elevation
- year
- tree-forming specie(s)
- aspect

The original locality names were used to locate sites, based on a query in the standard Norwegian topographic maps (NMA 2017). If locality names had changed, they were identified with the help of historical analogue maps. Some exceptions were made in areas with lower geographical coverage (for instance, the registrations originally by Finn Frost) and a total of seven registrations are missing the original aspect and marked 'not applicable' (NA).

**Definition for resampled/new TFLs**: TLs were defined as trees exceeding 2.5 m in height, measured vertically from the base, with one defined stem and a well-defined crown. This criterion was established to exclude shrubby forms characterised by dense growth. FLs were defined with a minimum of 15 trees, all fulfilling the definition of a tree and all with a minimum distance of 15 m between each tree within the same forested area.

**Uncertainty**: Several uncertainties were inherent in the process of resampling; however, the degree of uncertainty varied between the original dataset and the resampled/new dataset. Both datasets include two main uncertainties: i) elevational uncertainty and ii) spatial uncertainty. In terms of elevation, the uncertainty in the original data stems from the contemporary equipment and baseline maps used for calibrating aneroid barometers. In contrast, the elevation uncertainty in the resampled data, which is based on GPS measurements, is much lower (Table 2) and has undergone quality control (as described in the following sub-chapter). Regarding spatial uncertainty, the original data lack georeferenced coordinates. Consequently, the exact geographical locations for resampling remain uncertain in some municipalities and regions, particularly where multiple mountains share the same name. When no additional information was available to distinguish these mountain localities, they were excluded from further resampling efforts. Lastly, some uncertainty is found in the definitions from the original dataset, as the original authors used imprecise definitions (e.g. “..single-stemmed birch trees that exceed a man's height“, Table 1). Due to these inaccurate definitions, we used a more conservative definition (i.e. minimum tree height 2.5 m, minimum 15 trees in a forest with a maximum distance of 15 m between trees) to prevent overestimating TFL dynamics, while ensuring compatibility with the original TFL entries (Table 1).

### Quality control

Before the resampling process began, all fieldworkers spent time together calibrating their understanding of the definitions of a tree and a forest (according to the original authors) and the sampling procedure in general. During resampling, we used traditional aneroid barometers in addition to GPS, to test the quality of the original dataset. This approach allowed us to obtain a comparable estimate of the elevation uncertainty present in the original data, as the newly-collected data could be directly compared with the elevation measurements provided by the GPS (Table 2).

The elevation of all the resampled entries was also interpolated with a high-resolution Digital Elevation Model (DEM) in a Geographic Information System (GIS). Sufficient quality of the georeferenced coordinates was ensured by manual interpretation of updated aerial photos. Once the exact locations were established, the shadow of most trees along the treeline could be detected in high-resolution aerial photos no older than 5 years and available for all of Norway. All resampled TFLs were documented with photos (camera with GPS) and stored internally. To ensure adequately harmonised resampling between all the fieldworkers, 25% of the registrations were cross-checked by manually inspecting the attributes given by the photos. The photos provided evidence of presence only, thus allowing for the detection of only true and false positives.

## Geographic coverage

### Description

The TFLs have been resampled in five counties in Norway (Fig. 2). Parts of the datasets cover large mountain regions of south-central Norway (Trøndelag and Innlandet), whereas the rest of the dataset covers a fjord-valley region of western Norway (Vestland) and an east-west gradient of northern Norway (Nordland and Troms). Central Norway has the most entries, whereas the uttermost coastal mountains and the northernmost mountain regions (Finnmark) lack entries. The dataset has been collected in the uppermost parts of the boreal (FL) and the lower parts of the alpine (TL) bioclimatic regions (Bakkestuen et al. 2008).

### Coordinates

60°30'49'' and 68°58'10'' Latitude; 4°53'25'' and 19°15'57'' Longitude.

## Taxonomic coverage

### Description

The dataset presented in this paper includes occurrence data exclusively for mountain birch (Betula pubescens subsp. czerepanovii), which is also known by its two common synonyms: Betula pubescens subsp. tortuosa (Ledeb.) Nyman and Betula pubescens var. pumila (L.) Govaerts. In GBIF, the higher nomenclature was added to all records, from Kingdom to Subspecies. Misidentification of mountain birch (Betula pubescens subsp. czerepanovii) at the highest elevated locations is unlikely because it is predominantly the only tree-forming birch species present at the boreal-alpine ecotone in Norway.

### Taxa included

**Table:** 

Rank	Scientific Name	Common Name
subspecies	Betula pubescens subsp. czerepanovii	Mountain birch

## Traits coverage

The TFLs have been resampled to follow the definitions provided by the original authors as closely as possible (Table 1). Therefore, it is the location of a life form that has been georeferenced.

By adding GPS coordinates, the georeferenced resolution is now highly improved and the resampled TFLs are more precise, compared to the original georeferenced resolution. We thereby offer the same entries to be further resampled in the future.

The TL entries include mountain birch trees taller than 2.5 m and the FL entries include forested areas with at least 15 mountain birch trees (all fulfil positions less than 15 m apart).

In addition to structural traits (i.e. height, stem diameter) and topographical traits (i.e. slope, altitude), environmental traits, such as age class and condition, are also recorded.

## Temporal coverage

### Notes

**Figure 1.**
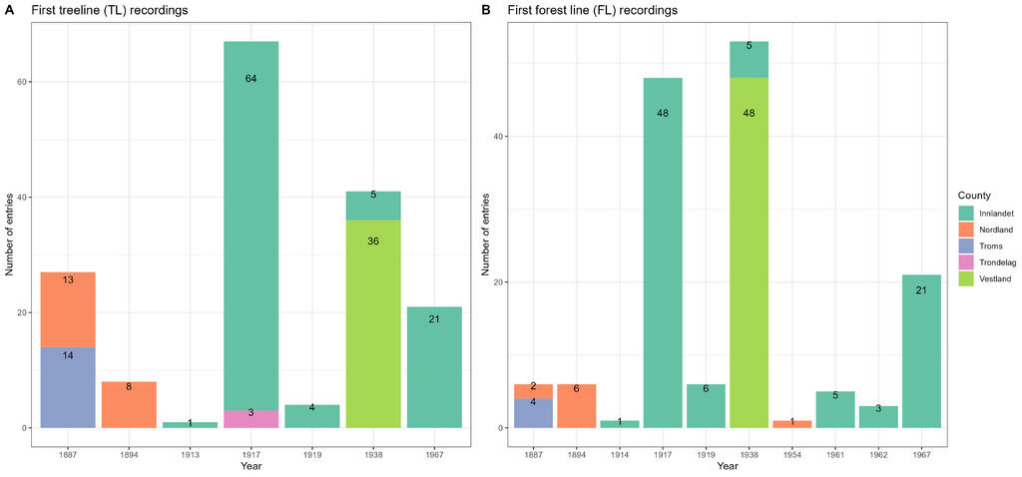
The historical registrations of treelines **(A)** and forest lines (TFLs) distributed in five counties in Norway **(B).**

**Figure 2.**
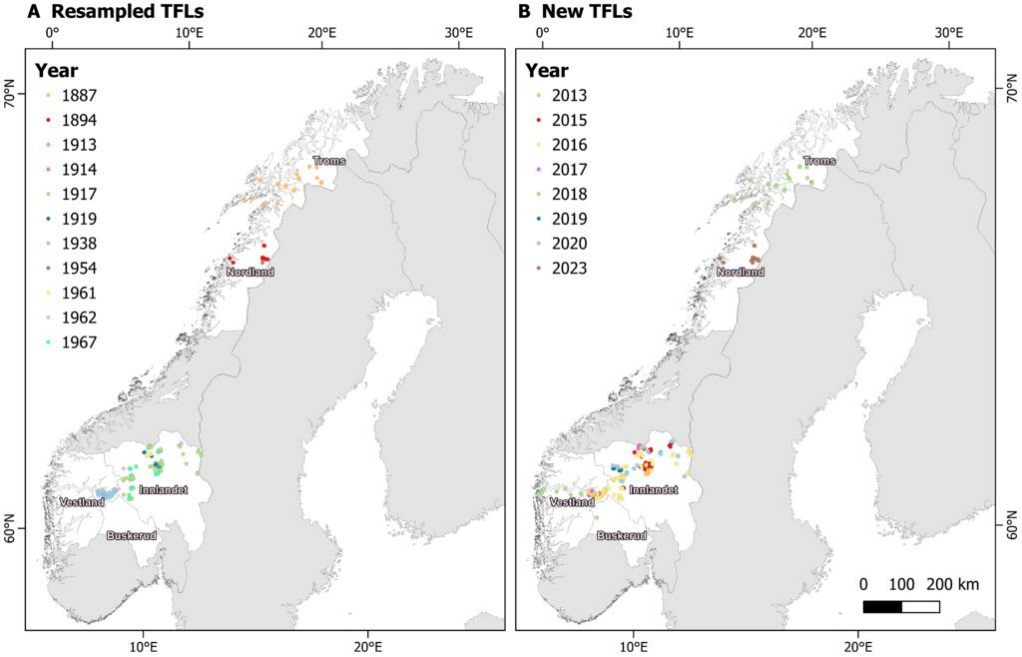
Distribution of tree- and forest lines (TFLs) in mainland Norway obtained through historical registrations **(A)** and through new sampling **(B)**.

The resampled data relate to five studies, first registered in 1887, 1894, 1913-14, 1917, 1919, 1938, 1954, 1961–62 and 1967 (Table 1). The same sites were resampled between 2013 and 2023. The temporal coverage of TLs varies from 27 entries in the year 1887, eight entries in 1894, one entry from 1913–1914, 67 entries from 1917, four entries from 1919, 41 entries from 1938 and 21 entries from 1967 (Fig. 1A). The temporal coverage of FLs varies from six entries in the year 1887, six entries in 1894, one entry from 1913–1914, 48 entries from 1917, six entries from 1919, 53 entries from 1938, one entry from 1954, eight entries from 1961–62 and 21 entries from 1967 (Fig. 1B).

## Usage licence

### Usage licence

Other

### IP rights notes

Creative Commons Attribution 4.0 International (CC-BY-4.0)

## Data resources

### Data package title

Sampling of highest Betula pubescens subsp. czerepanovii treelines and forest lines in Norway

### Resource link

https://doi.org/10.15468/ua2sdc

### Alternative identifiers

https://www.gbif.org/dataset/0a46c2f5-1d7c-49e9-bfd2-7065db24e168

### Number of data sets

1

### Data set 1.

#### Data set name

Sampling of highest Betula pubescens subsp. czerepanovii treelines and forest lines in Norway

#### Data format

Darwin Core Archive (DwC-A)

#### Character set

UTF-8

#### Download URL

https://ipt.gbif.no/archive.do?r=tf-fl-norge&v=1.8

#### Description

The dataset is structured in Darwin Core (DwC) format, utilising the Event Core and Occurrence extension to organise and standardise the data. Each event represents a collection of treeline or forest line observations made at a specific location, encapsulating both historical and modern records. Historical observations, which typically include only altitude and the name of the location, are grouped with newer registrations that feature precise GPS coordinates. This approach ensures that all observations associated with a given site are grouped logically, facilitating temporal comparisons and allowing for comprehensive analysis of changes over time, even when precise spatial data is unavailable for older records.

To enhance data richness and address limitations in the DwC schema, additional information is stored in the dynamicProperties field in JSON format.

**Data set 1.** 

Column label	Column description
eventID (Event core)	Unique identifier for an event.
eventDate (Event core)	ISO 8601 compliant date interval of when the event occurred.
sampleSizeValue (Event core)	Number of years over which the event occurred.
sampleSizeUnit (Event core)	Describes event_sampleSizeValue units. Years in our case.
country (Event core)	The name of the country in which the location occurs.
countryCode (Event core)	ISO 3166 compliant country code.
stateProvince (Event core)	Name of the province (`Fylke`) where the even occurred.
municipality (Event core)	Name of the municipality (`Kommune`) where the even occurred.
eventID (Occurrence extension)	A reference of the dwc:Event encompassing the sampling location.
basisOfRecord (Occurrence extension)	The specific nature of the data record.
dynamicProperties (Occurrence extension)	This field includes the following attributes in JSON format: Forest_Tree: Specifies whether the observation pertains to a treeline (TL) or forest line (FL). Topmost: Indicates whether the observation is the actual highest altitude registration (TOP) or an additional registration at the boundary (ADD). Rep_New: Distinguishes between new observations (NEW) and repetitions of historical records (REP). treeHeight: Captures tree height in metres. condition: Reflects the progress of the forest in the area using values of +1, 0, or −1, where 0 denotes a stable boundary, +1 suggests significant new growth indicating upward progression and −1 signals visible die-off and potential retreat of the boundary age: Provides an estimated age of the registered tree. aspect: Records the aspect of the slope (N, NE, E, SE, S, SW, W, NW) where the observation was made.
occurrenceID (Occurrence extension)	An identifier for the occurrence.
recordedBy (Occurrence extension)	Names of the person(s) who has recorded data.
recordedByID (Occurrence extension)	Unique identifiers of the person(s) who has recorded data.
occurrenceStatus (Occurrence extension)	A statement about the presence or absence of a dwc:Taxon at a dcterms:Location.
year (Occurrence extension)	The four-digit year in which the Event occurred, according to the Common Era Calendar.
verbatimLocality (Occurrence extension)	A textual description of the place name as it was written when recorded.
locality (Occurrence extension)	Most up-to-date name of the locality
maximumElevationInMetres (Occurrence extension)	The upper limit of the range of elevation (altitude above sea level) of the location, in metres.
decimalLatitude (Occurrence extension)	The geographic latitude (in decimal degrees, using the spatial reference system given in geodeticDatum) of the geographic centre of a location.
decimalLongitude (Occurrence extension)	The geographic longitude (in decimal degrees, using the spatial reference system given in geodeticDatum) of the geographic centre of a location.
geodeticDatum (Occurrence extension)	The ellipsoid, geodetic datum or spatial reference system (SRS), upon which the geographic coordinates given in dwc:decimalLatitude and dwc:decimalLongitude are based.
coordinateUncertaintyInMetres (Occurrence extension)	The coordinate uncertainty of the specific locality given in metres.
verbatimCoordinates(Occurrence extension)	The verbatim original spatial coordinates of the dcterms:Location.
verbatimCoordinateSystem (Occurrence extension)	UTM grid zone designation (e.g. ‘33W’) recorded in the field as the original spatial reference system.
scientificName (Occurrence extension)	The full scientific species name, including authorship information.
kingdom (Occurrence extension)	The full scientific name of the kingdom in which the dwc:Taxon is classified.
taxonRank (Occurrence extension)	The taxonomic rank of the most specific name in the dwc:scientificName.

## Figures and Tables

**Table 1.** Reference to the original author, year of the original sampling, number of TL and/or FL entries and definition used by the original sampling author.

**Original sampling author (reference)**	**Sampling year**	**Treelines**	**Forest lines**	**Definition (TL and/or FL)**
Norman (1894)	1887	27	6	The highest elevation above sea level at which upright, single-stemmed birch trees exceed a man's height.
Norman (1894)	1894	8	6	
Resvoll-Holmsen (1914)	1913	1	0	The highest elevation above sea level at which upright, single-stemmed birch trees exceed human height (after Norman (1894)). The upper limit for continuous forest.
Resvoll-Holmsen (1914)	1914	0	1	
Resvoll-Holmsen (1918)	1917	67	48	
Resvoll-Holmsen (1920)	1919	4	6	
Ve (1940)	1938	41	53	Uppermost individuals of single trees, at least as tall as a man (after Norman (1894)) Continuous clusters of forest of at least 14-15 individuals.
Frost (1954)	1954	0	1	"The birch forest line". No precise definition.
Aas (1964)	1961	0	5	Uppermost individuals of single trees, at least as tall as a man (after Ve (1940)). Continuous clusters of forest of at least 15 individuals, with a minimum height of 2.5 metres and a maximum distance of 30 metres between the trees.
Aas (1964)	1962	0	3	
Aas (1969)	1967	21	21	
**Total**		**169**	**150**	

**Table 2.** Elevation uncertainty measures are given as mean and standard deviation of the differences in altitude measured by GPS and barometer, GPS and the Digital Elevation Model (DEM) and barometer and DEM.

**Comparisons**	**Mean (m)**	**Standard deviation (m)**
DEM - GPS	0.11	6.47
Barometer - DEM	-2.06	21.03
GPS - barometer	2.15	19.39
